# Reducing Systematic Uncertainty in Computed Redox Potentials for Aqueous Transition-Metal-Substituted Polyoxotungstates

**DOI:** 10.1021/acs.inorgchem.3c01115

**Published:** 2023-07-25

**Authors:** Jake A. Thompson, Rebeca González-Cabaleiro, Laia Vilà-Nadal

**Affiliations:** † School of Chemistry, University of Glasgow, Glasgow G12 8QQ, U.K.; ‡ Department of Biotechnology, Delft University of Technology, Delft 2628 CD, Netherlands

## Abstract

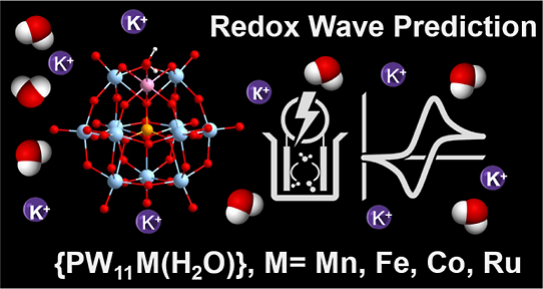

Polyoxometalates have attracted significant interest owing to their structural diversity, redox stability, and functionality at the nanoscale. In this work, density functional theory calculations have been employed to systematically study the accuracy of various exchange–correlation functionals in reproducing experimental redox potentials, *U*^0^_Red_ in [PW_11_M(H_2_O)O_39_]^*q*−^ M = Mn(III/II), Fe(III/II), Co(III/II), and Ru(III/II). *U*^0^_Red_ calculations for [PW_11_M(H_2_O)O_39_]^*q*−^ were calculated using a conductor-like screening model to neutralize the charge in the cluster. We explicitly located K^+^ counterions which induced positive shifting of potentials by > 500 mV. This approximation improved the reproduction of redox potentials for K_*x*_[XW_11_M(H_2_O)O_39_]^*q*−*x*^ M = Mn(III/II)/Co(III/II). However, uncertainties in *U*^0^_Red_ for K_*x*_[PW_11_M(H_2_O)O_39_]^*q*−*x*^ M = Fe(III/II)/Ru(III/II) were observed because of the over-stabilization of the ion-pairs. Hybrid functionals exceeding 25% Hartree–Fock exchange are not recommended because of large uncertainties in Δ*U*^0^_Red_ attributed to exaggerated proximity of the ion-pairs. Our results emphasize that understanding the nature of the electrode and electrolyte environment is essential to obtain a reasonable agreement between theoretical and experimental results.

## Abstract 2

Density functional theory calculations have been employed to assess the accuracy of exchange−correlation functionals in reproducing experimental redox potentials, *U*^0^_Red_ in [PW_11_M(H_2_O)O_39_]^*q*−^ M = Mn(III/II), Fe(III/II), Co(III/II), and Ru(III/II). Furthermore, we have explicitly located (K^+^) counterions, rendering our systems’ charge neutral forming K_*x*_[XW_11_M(H_2_O)O_39_]^*q*−*x*^ (*x* = 4 or 5) and assessing the effect of computed *U*^0^_Red_ values.

## Introduction

**Figure 1:**
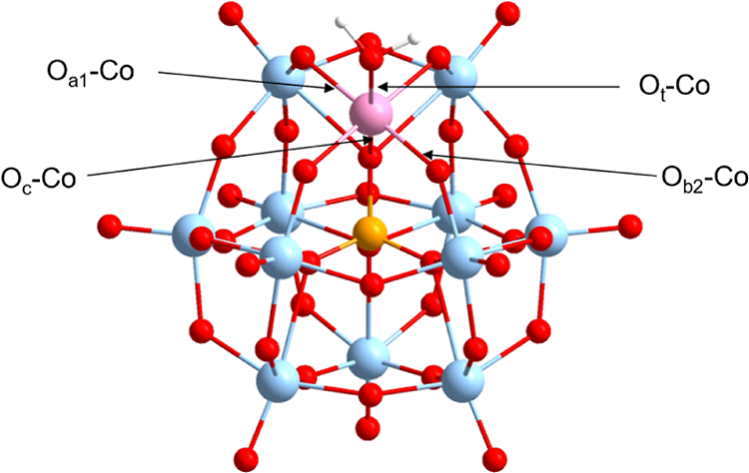
Schematic representation for the cobalt(II)-substituted Keggin [PW_11_Co(H_2_O)O_39_]^*q*−^ anion. Colors corresponding to W = cyan, O = red, P = orange, and Co = pink.

**Scheme 1:**
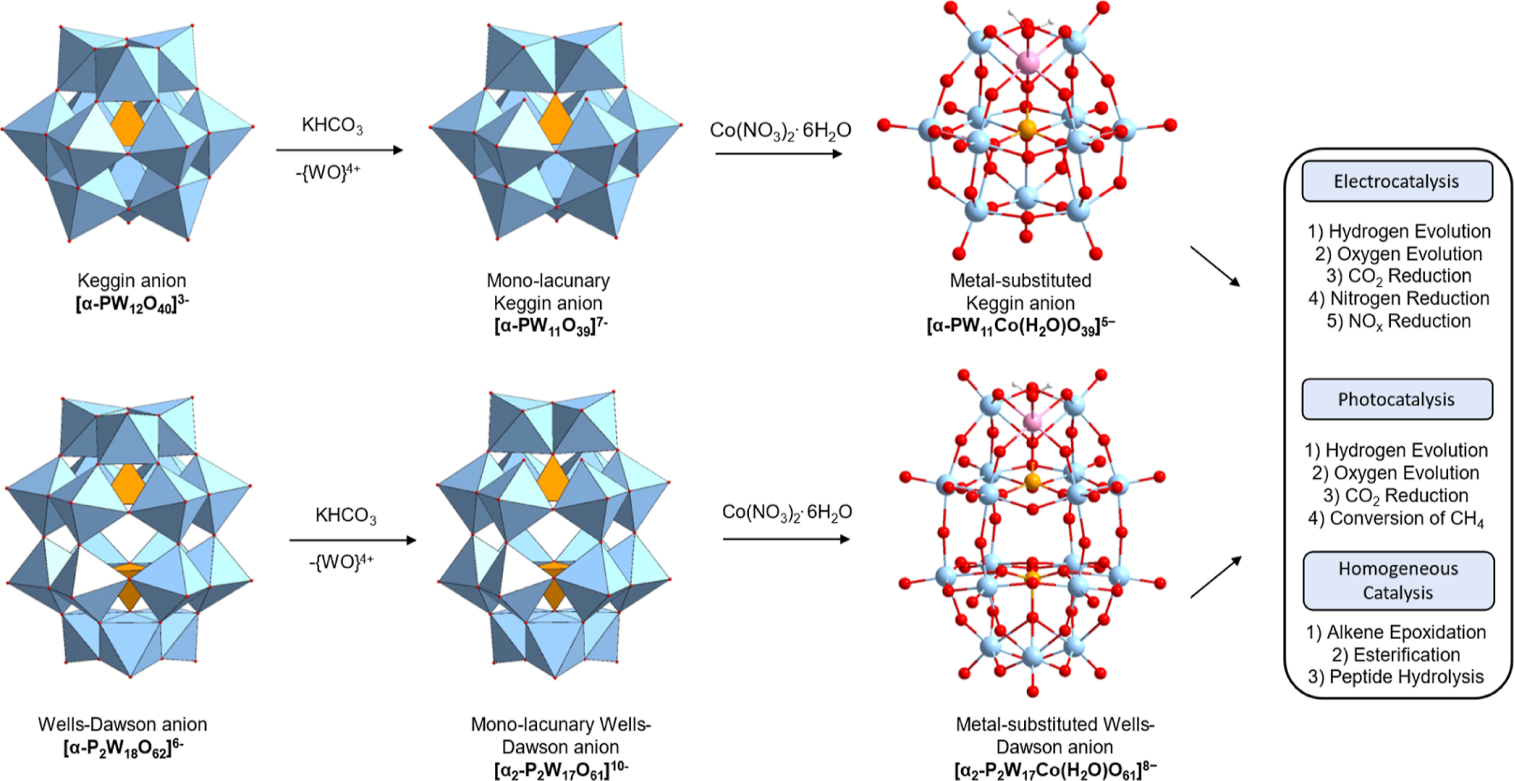
General Synthetic Strategy for Obtaining Co(II)-Substituted Keggin [α-PW_11_Co(H_2_O)O_39_]^5–^ and Wells–Dawson [α_2_-P_2_W_17_Co(H_2_O)O_61_]^8–^ Derivatives

Polyoxometalates (POMs) are a large group of discrete, polynuclear metal-oxo clusters comprising early transition-metal (addenda) and oxide atoms.^1−5^ Addenda atoms are fully oxidized to d^0^ electron configurations capable of forming various topologies employing {MO_*x*_} as the principal building block.^1−5^ Poly-oxo clusters are formed from the acidification of aqueous molybdate or tungstate oxoanions.^6,7^ Partial hydrolysis of poly-oxo clusters, achieved through the controlled addition of base produces lacunary clusters. These complexes formally lose one or several M = O vertices and possess reactive cavities with high charge density around the defective region due to the negatively charged oxygen ligands.^8^ The defective region can react with transition-metal cations forming a new class of compounds, an example shown are mono-transition-metal-substituted polyoxotungstates, [XW_11_M(L)O_39_]^*q*−^ (X = e.g., P(V), Si(IV), L = H_2_O, DMSO, etc.)—see Table 1.^8^ Conventionally, hexacoordinate transition-metals are introduced so the sixth coordination site are occupied by solvent ligands from the local environment—see Figure 1. These structures have attracted significant interest as single atom catalysts^9^ in fields including water oxidation,^10,11^ carbon dioxide reduction,^12,13^ and nitrogen activation (Scheme 1).^14^

**Table 1:** Selected Mono-Transition-Metal-Substituted Keggin, [PW_11_M(L)O_39_]^*q*−^, and Wells–Dawson, [P_2_W_17_M(L)O_61_]^*q*−^, Derivatives^a^

M, [XW_11_M(L)O_39_]^*q*−^	XO_4_, *q* = 4	XO_4_, *q* = 5	XO_4_, *q* = 6
Sc	^a^PO_4_^15^		
Cr	^a^PO_4_^16,17^	^a^SiO_4_^17,18^	
Ti		^b^PO_4_^19^	
Mn	^a^PO_4_^20^	^a^PO_4_^20^^a^SiO_4_^20^^a^GeO_4_^20^	^a^BO_4_^20^^a^SiO_4_^20^^a^GeO_4_^20^
Fe	^a^AsO_4_^21^^a^PO_4_^21^	^a^PO_4_^21^^a^SiO_4_^21^^a^GeO_4_^21^	^a^SiO_4_,^21^^a^GeO_4_^21^
Co		^a^PO_4_^22^	^a^SiO_4_^23^
Ru	^a,c^PO_4_^24^	^a^PO_4_^25^^a,c^SiO_4_^26^^a,c^GeO_4_^27^	
Rh	^a^PO_4_^28^		^d^SiO_4_^29^
Ir	^a^PO_4_^30^		
Ni		^a^PO_4_^18^	^a^SiO_4_^31^
Cu		^a^PO_4_^18,20^	^a^SiO_4_^20^^a^GeO_4_^20^
Zn		^a^PO_4_^18^	

M, [X_2_W_17_M(L)O_61_]^*q*−^	XO_4_, *q* = 6	XO_4_, *q* = 7	XO_4_, *q* = 8
Cr		^a^PO_4_^17^	
Ti			^e^PO_4_^32^
Mn		^a^AsO_4_^20^^a^PO_4_^20,33^	^a^AsO_4_^20^^a^PO_4_^20^
Fe		^a^PO_4_^33^	
Co			^a^PO_4_^33,34^
Ru		^a,b^PO_4_^35^	^a,b^PO_4_^35^
Ir	^a^PO_4_^36^		
Ni			^a^PO_4_^33^
Cu			^a^AsO_4_^a^PO_4_^33^
Zn			^a^PO_4_^37^

a Note: ^a^H_2_O, ^b^O, ^c^C_2_H_6_OS, ^d^Cl^–^, ^e^O_2_.

Early computational work on POMs have primarily focused on charged molecules using implicit solvation models.^38^ In this regard, Poblet and co-workers reported the relative stability of rotational isomers of Keggin heteropolyanions, α/β-[XM_12_O_40_]^*q*−^.^39^ These calculations were performed using the local-density approximation functional coupled with Vosko–Wilk–Nusair parametrization, in the gas phase.^39^ The enhanced stability of the α-[XM_12_O_40_]^*q*−^ isomer was attributed to the higher energy of the lowest occupied molecular orbital.^39^ However, it was shown that four-times-reduced clusters [PW_12_O_40_]^7–^ and [SiMo_12_O_40_]^8–^ demonstrated enhanced stability (ca. 0.4 eV) toward the β-isomer.^39^ Later, Zhang and co-workers emphasized the importance of implicit solvation for reproducing experimental geometries.^40^ Several generalized gradient approximation (GGA) functionals were tested, in which Perdew–Burke–Ernzerhof (PBE) and Becke 1988 exchange and Perdew 86 (BP86) methods provided the closest description to the experimental geometries.^40^ However, GGA functionals often poorly describe electron delocalization as previously reported by Poblet and co-workers.^41^ Their work showed GGA functionals incorrectly localized an additional electron at the belt region in mono-substituted Wells–Dawson [P_2_W_17_MO_62_]^*q*−^ (M = V, Mo) anions.^41^ On the other hand, employment of hybrid methods, such as B3LYP or M05 correctly localized the electron.^41^ However, only B3LYP [20% Hartree–Fock (HF) exchange] gave the correct ordering and relative reduction energies with respect to experimental measurements.^41^

Quantum chemical calculations reproducing redox potentials in POMs have been reported without accounting for electrolyte environment and counterions, using implicit solvation models. Aparicio and co-workers computed the tungsten redox waves for mono-substituted Keggin, [XMW_11_O_40_]^*q*−^ (M = W, Mo, V, Nb, and Ti) using a conductor–like screening mode.^42^ This simplification produced large uncertainties in absolute reduction potential. However, shifts in potential with respect to the Keggin [XM_12_O_40_]^*q*−^ anion were reproduced with corresponding experimental data.^42^ Recently, Rösch and co-workers reported redox potentials for the tri-Mn-substituted Keggin in which counterions were explicitly located onto the surface of POM to neutralize the system.^43^ The authors employed several exchange–correlation (*x*–*c*) functionals with the increase in contributions of HF exchange (0% PBE, 10% TPSSh, 20% B3LYP, 25% PBE0) which produced redox potentials that increase (in that order) with the exact exchange.^43^ The closest agreement with experimental literature was shown for the GGA–PBE functional attributed to fortuitous error cancellations. By contrast, hybrid exchange–functionals overestimated experimental potentials by 0.6–1.0 V.^43^ This work was extended to include explicit treatment of water molecules derived from molecular dynamics simulations.^44^ The inclusion of explicit solvation significantly improved computed redox potentials; however, its contributions were out-weighted by the choice of exchange–correlation functional.^44^ In this work, optimal reproduction of experimental literature were obtained using the B3LYP method.^44^ Recently, Falbo and Penfold reported the influence of the self-interaction error could be reduced by neutralizing the charged species by incorporating counterions into the system.^45^ The authors reported excellent agreement, within 0.1 V of the experimental literature for the first redox wave in Na_3_[SiW_12_O_40_].^45^

In this work, we have performed systematic density functional theory (DFT) calculations to study the redox properties of [PW_11_M(H_2_O)O_39_]^*q*−^ M = Mn(III/II), Fe(III/II), Co(III/II), and Ru(III/II) and their potassium salts. For achieving adequate molecular geometries, we present a structural benchmark for the cobalt(II)-substituted Keggin against the crystallographic structure.^46^ Thereafter, we explore the challenges in computing redox potentials and provide an insight into the geometric and electronic factors controlling it.

## Computational Details

All computational results were obtained using the ARCHIE–WeSt high-performance computer based at the University of Strathclyde. DFT calculations were performed using the Amsterdam Modelling Suite (AMS 2020.1) package.^47^ In this work, several classes of exchange–correlation (*x*–*c*) functionals were employed, which include (i) generalized gradient approximation (GGA); (ii) hybrid; and (iii) range-separated hybrid functionals. GGA functionals considered were as follows: (i) PBE;^48^ (ii) Perdew–Wang (PW91);^49^ and (iii) Becke 1988 exchange and Perdew 86 (BP86).^50,51^ The hybrid *x*–*c* functionals considered were as follows: (i) Becke, 3-parameter, Lee–Yang–Parr (B3LYP*,^52^ B3LYP^53^); (ii) PBE0;^54^ and (iii) Becke’s half-and-half (BH&H).^55^ Hybrid functionals were selected on their contributions of HF exchange (15% B3LYP*, 20% B3LYP, 25% PBE0, and 50% BH&H). The ωB97X method was selected as the range-separated hybrid functional.^56^ We employed Slater basis sets comprising the following: (i) triple-ζ polarization (TZP); (ii) triple-ζ plus polarization (TZ2P); and (iii) quadruple-ζ plus polarization (QZ4P).^57,58^ Relativistic corrections were included by means of the zeroth order regular approximation formalism.^59^ The effects of aqueous solvent were approximated by using the conductor-like screening model, as implemented by AMS.^60^ For open shell molecules, unrestricted Kohn–Sham (UKS) theory was implemented, while restricted Kohn–Sham (RKS) theory was employed for closed shell systems. All harmonic vibrational frequencies were calculated using PBE coupled with the TZP basis set. The calculation of Gibbs free energies for hybrid-optimized systems were corrected by using the zero-point energies and entropic components obtained from GGA–vibrational frequencies—see eq 1.0.
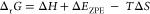
1.0Herein, Δ*H* equates to the enthalpic component; Δ*E*_ZPE_ is the difference in zero-point energy, and *T*Δ*S* is the entropic component, under standard conditions, *T* = 298.15 K, *P* = 1.0 atm. The entropic and zero-point terms were computed using harmonic vibrational frequencies. Cramer and co-workers reported the free energy change for the standard hydrogen electrode (SHE) half-reaction (1/2H_2_ → H^+^ + e^–^) equates to 4.24 eV.^61^ This was used an external reference for all computed potentials.

To evaluate the discrepancy of the calculated versus crystallographic geometries, we employed mean absolute error (MAE), mean signed error (MSE), and standard deviation (STD) calculated using
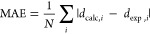
1.1
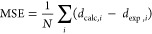
1.2
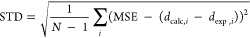
1.3where *d*_calc_ and *d*_exp_ are the calculated and experimental bond distances, respectively.

## Results and Discussion

### Structural Benchmark-Anionic Model

The selection of the exchange–correlation (*x*–*c*) functional and basis set and its ability to precisely describe the system of interest is a prerequisite for DFT studies. To assess the accuracy of *x*–*c* functionals and basis sets on the structural optimization of [PW_11_Co(H_2_O)O_39_]^*q*−^, MSE, and MAE were used to compare against the crystallographic structure, reported by Cavaleiro and co-workers.^46^ Four types of oxygen atoms were examined: O_c_ and O_t_ which correspond to the heteroatom–oxygen and terminal–oxygen atoms, while O_a1_ and O_b2_ denote bridging (equatorial) oxygens bound to the newly incorporated transition-metal—see Figure 1.

**Figure 2:**
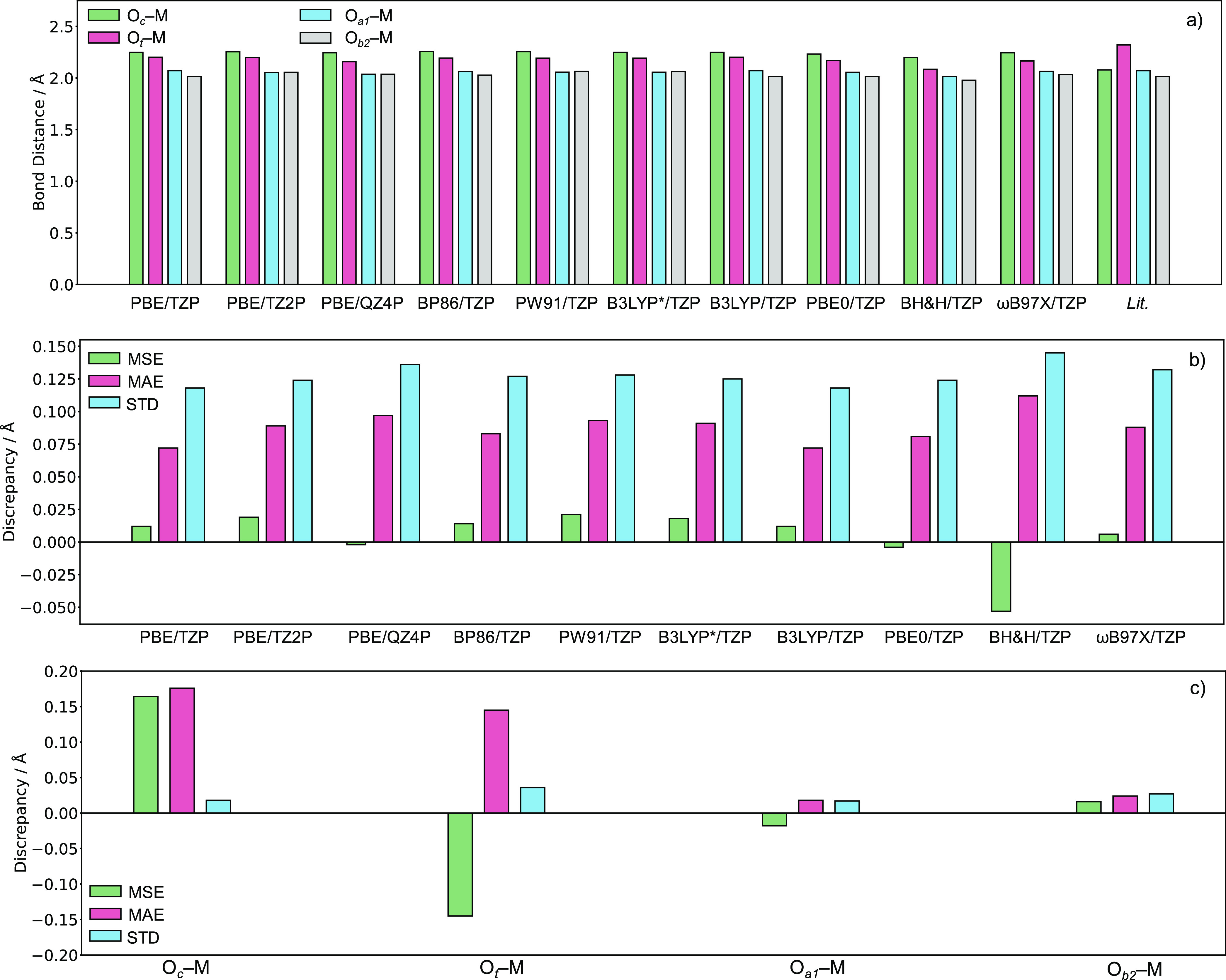
(a) Selected structural parameters for the cobalt(II)-substituted Keggin [PW_11_Co(H_2_O)O_39_]^*q*−^ anion referenced against the crystallographic structure taken from Cavaleiro and co-workers.^46^ (b) MSE, MAE, and STD calculated for all applied functionals and basis sets. (c) MSE, MAE, and STD calculated for four types of metal–oxygen interactions. All bond distances are reported in Å.

Figure 2a presents results for the selected structural parameters of [PW_11_Co(H_2_O)O_39_]^*q*−^ employed to assess the accuracy of applied functionals and basis sets. As is evident, equilibrium geometries in [PW_11_Co(H_2_O)O_39_]^*q*−^ were generally well described by all methods, rarely exceeding discrepancies of 0.18 Å. Figure 2b showed for systems with the increase in HF exchange, the magnitude of MSE progressively increased. Of particular note, hybrid functionals exceeding 20% HF exchange commenced underestimating equilibrium geometries, shown by MSE. For BH&H (50% exchange), MSE of ca. −0.05 Å was attributed to the underestimation of O_t_–Co, O_a1_–Co, and O_b2_–Co by 0.236, 0.059, and 0.035 Å, respectively. Figure 2c reported calculated errors with respect to the selected bond. Herein, the primary source of error was the reproduction of the heteroatom–oxygen and terminal–oxygen bonds: O_c_–Co and O_t_–Co. The crystallographic structure of [PW_11_Co(H_2_O)O_39_]^*q*−^ illustrates the magnitude of O_*t*_–Co surpasses O_*c*_–Co, reporting distances of 2.321 and 2.079 Å, respectively.^46^ However, our models have inverted this observation, in which the magnitude of O_c_–Co exceeds O_t_–Co. Across all methods, the equatorial distances were accurately described with computed errors rarely exceeding 0.02 Å. The equatorial parameters were insensitive across all applied functionals and basis sets, reporting a range of 0.059 and 0.085 Å for O_a1_–Co and O_b2_–Co, respectively.

**Figure 3:**
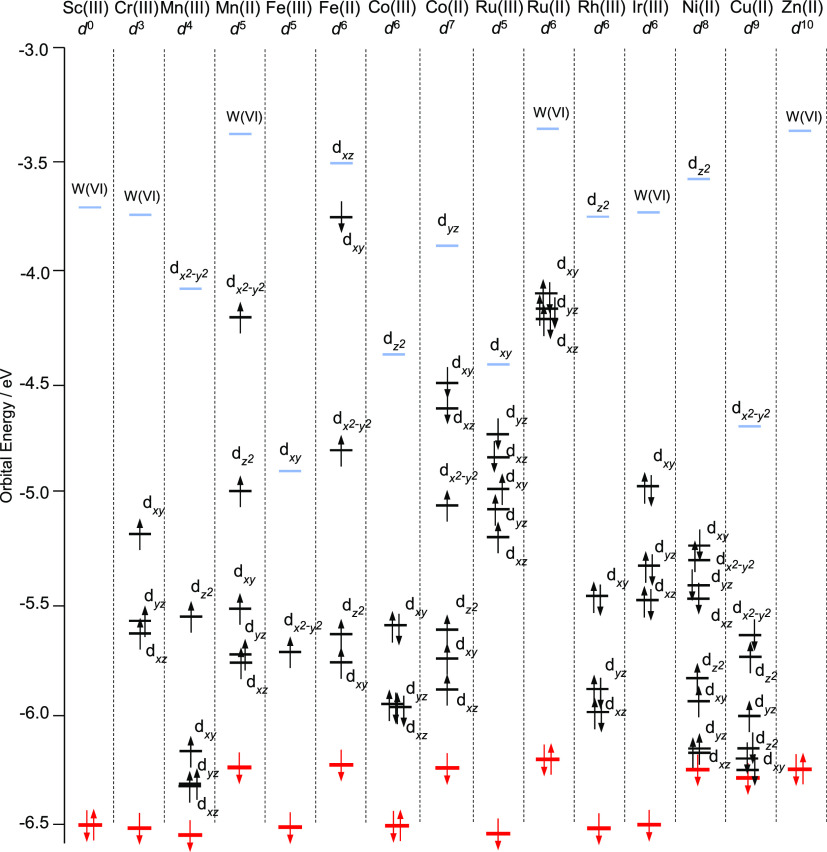
Schematic molecular orbital diagram for [PW_11_M(H_2_O)O_39_]^*q*−^ systems, optimized using the PBE/TZP level of theory. Herein, closed shell [PW_11_M(H_2_O)O_39_]^*q*−^ systems were computed with the RKS theory—distinguished by spin-paired orbitals. Open shell [PW_11_M(H_2_O)O_39_]^*q*−^ systems were computed with UKS theory and are distinguished by separate spin-up and spin-down orbitals. Colors correspond to red = O2(p), blue = W, and black = transition-metal. All orbital energies reported in eV.

The electronic structure of a fully oxidized POM consists of two identifiable bands: (i) an oxo-band comprising occupied oxo-ligands; (ii) and a metallic band encompassing unoccupied addenda orbitals. Figure 3 depicts the frontier molecular orbitals of [PW_11_M(H_2_O)O_39_]^*q*−^ anions, optimized using the PBE/TZP level of theory. Herein, orbitals associated with the newly incorporated transition-metal are inserted between the occupied oxo and unoccupied addenda band.

Cyclic voltammetric experiments have explored the redox behavior for [PW_11_M(H_2_O)O_39_]^*q*−^ M = Mn(III/II),^20^ Fe(III/II),^21^ Co(III/II),^23,24^ and Ru(III/II).^24,26,27^Figure 3 and Table-S1 will be used to account for modifications to molecular geometries of [PW_11_M(H_2_O)O_39_]^*q*−^ [M = Mn(II), Fe(II), Co(II), and Ru(II)], under electrochemical processes. One-electron oxidation of [PW_11_Mn(H_2_O)O_39_]^5–^ induced constriction of O_a1_–Mn and O_b2_–Mn by 0.197 and 0.125 Å, respectively. Oxidation of [PW_11_Mn(H_2_O)O_39_]^5–^ removes σ*(O_a1_–M) and σ*(O_b2_–M) interactions derived from the overlap of O2p_*x*_ or O2p_y_ orbitals and d_*x*^2^–*y*^2^_ orbitals. One-electron oxidation of [PW_11_M(H_2_O)O_39_]^*q*−^ (M = Fe(II) or Ru(II)) had no significant impact to molecular geometry. One-electron oxidation of [PW_11_Co(H_2_O)O_39_]^5–^ constricted O_c_–M by 0.284 Å attributed to the removal of σ*(O_c_–M) derived from the overlap of O2p_*z*_ and d_*z*^2^_ orbital. Herein, [PW_11_Co(H_2_O)O_39_]^4–^ possesses the electron configuration: (d_*xz*_)^2^(d_*yz*_)^2^(d_*xy*_)^2^ which are (predominantly) non-bonding. Previous work modeling redox processes of POMs have been calculated using the reduction energy (RE) which assumes entropic and vibrational contributions are negligible.^62^ However, the significance of σ*(O_c_–M), σ*(O_a1_–M), and σ*(O_b2_–M) interactions emphasize the importance of enthalpic, entropic, and zero-point contributions for redox modeling of [PW_11_M(H_2_O)O_39_]^*q*−^ [M = Mn(II) and Co(II)] anions.

### Computation of Redox Potentials-Anionic Model

**Figure 4:**
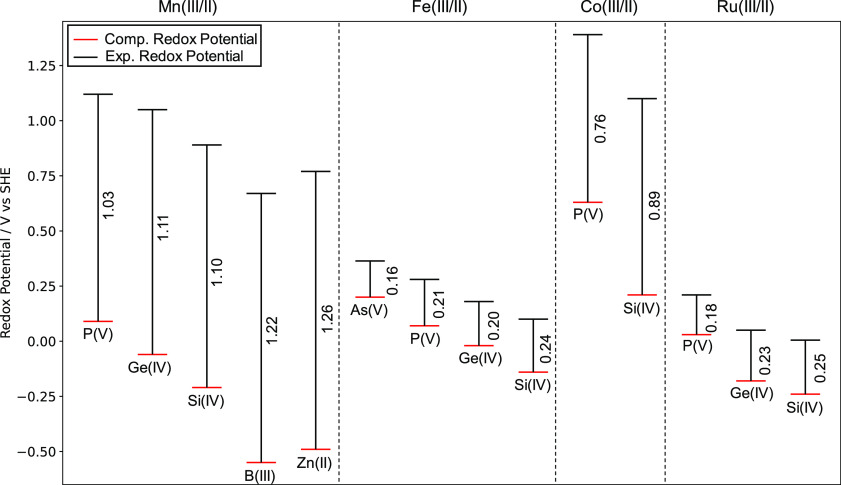
Redox potentials, *U*^0^_Red_ vs SHE, for Mn(III/II), Fe(III/II), Co(III/II), and Ru(III/II) couple present in [XW_11_M(H_2_O)O_39_]^*q*−^; X = As(V), Si(IV), Ge(IV), B(III), and Zn(II). All potentials were calculated using the PBE/TZP level of theory. Experimental potentials were obtained from Mn(III/II),^20^ Fe(III/II),^21^ Co(III/II),^23,24^ and Ru(III/II).^24,26,27^

Redox potentials, *U*^0^_Red_ versus SHE, for Mn(III/II), Fe(III/II), Co(III/II), and Ru(III/II) couples present in [XW_11_M(H_2_O)O_39_]^*q*−^; X = As(V), Si(IV), Ge(IV), B(III), and Zn(II) are reported in Figure 4. Computation of *U*^0^_Red_ for [PW_11_M(H_2_O)O_39_]^*q*−^ M = Fe(III/II)/Ru(III/II) closely reproduced the literature within ca. 0.2 V. By contrast, computed values of *U*^0^_Red_ for [PW_11_M(H_2_O)O_39_]^*q*−^ M = Mn(III/II)/Co(III/II) were notably underestimated by ca. 0.8—1.0 V. The discrepancy between DFT and experimental potential, *U*^0^_Err__or_ increased with overall anionic charge of [XW_11_M(H_2_O)O_39_]^*q*−^; attributed to the increasing contribution of the self-interaction error. For example, [ZnW_11_Mn(H_2_O)O_39_]^7–/8–^ produced *U*^0^_Error_ = 1.26 V superseding [PW_11_Mn(H_2_O)O_39_]^4–/5–^ by 0.23 V.

**Figure 5:**
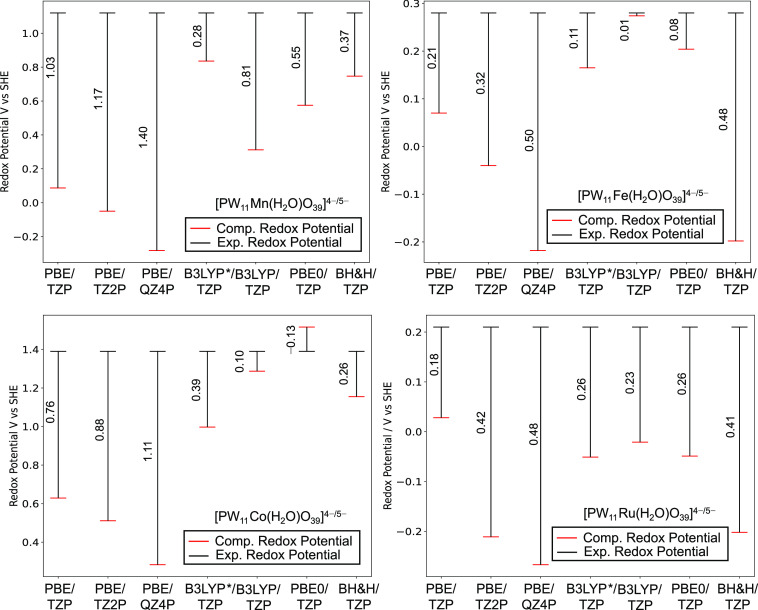
Redox potentials, *U*^0^_R__ed_ vs SHE, for Mn(III/II), Fe(III/II), Co(III/II), and Ru(III/II) redox couples in [PW_11_M(H_2_O)O_39_]^*q*−^. Experimental potentials were obtained from Mn(III/II),^20^ Fe(III/II),^21^ Co(III/II),^23,24^ and Ru(III/II).^24,26,27^

The effect of applied exchange–correlation functional and basis set was assessed for the Mn(III/II), Fe(III/II), Co(III/II), and Ru(III/II) couples in [PW_11_M(H_2_O)O_39_]^*q*−^—see Figure 5. The employment of larger basis sets (TZ2P, QZ4P) proved inferior in reproducing experimental potentials, with respect to triple-ζ + polarization (TZP) basis sets. For example, *U*^0^_Error_ for [PW_11_Mn(H_2_O)O_39_]^*q*−^ increased from 1.03 to 1.40 V (Δ*U*^0^_Error_ = 0.37 V) for TZP and QZ4P basis sets, respectively.

Generally, hybrid *x*–*c* functionals outperformed GGA methodologies by reducing *U*^0^_E__rror_ for M(III/II) redox couples. This is evidenced by *U*^0^_Error_ in [PW_11_Mn(H_2_O)O_39_]^*q*−^ which notably decreased from 1.03 to 0.28 V (Δ*U*^0^_E__rror_ = 0.75 V) by incorporating 15% HF exchange (B3LYP*/TZP). However, the optimal *x*–*c* functional was not consistent across all M(III/II) couples. For example, Fe(III/II), Co(III/II), and Ru(III/II) couples were optimally reproduced with 20% HF exchange (B3LYP/TZP) calculating *U*^0^_Error_ of 0.01, 0.10, and 0.23 V respectively. Thus far, *U*^0^_Red_ for [PW_11_Mn(H_2_O)O_39_]^*q*−^ were calculated without neutralizing their charge, and therefore, large discrepancies associated with the self-interaction error are expected for these calculations. Previous work showed explicitly locating counterions on the surface of the POM, rendering the system charge neutral can reduce this systematic error.^44^

### Computation of Redox Potentials-Charge Neutral Model

**Figure 6:**
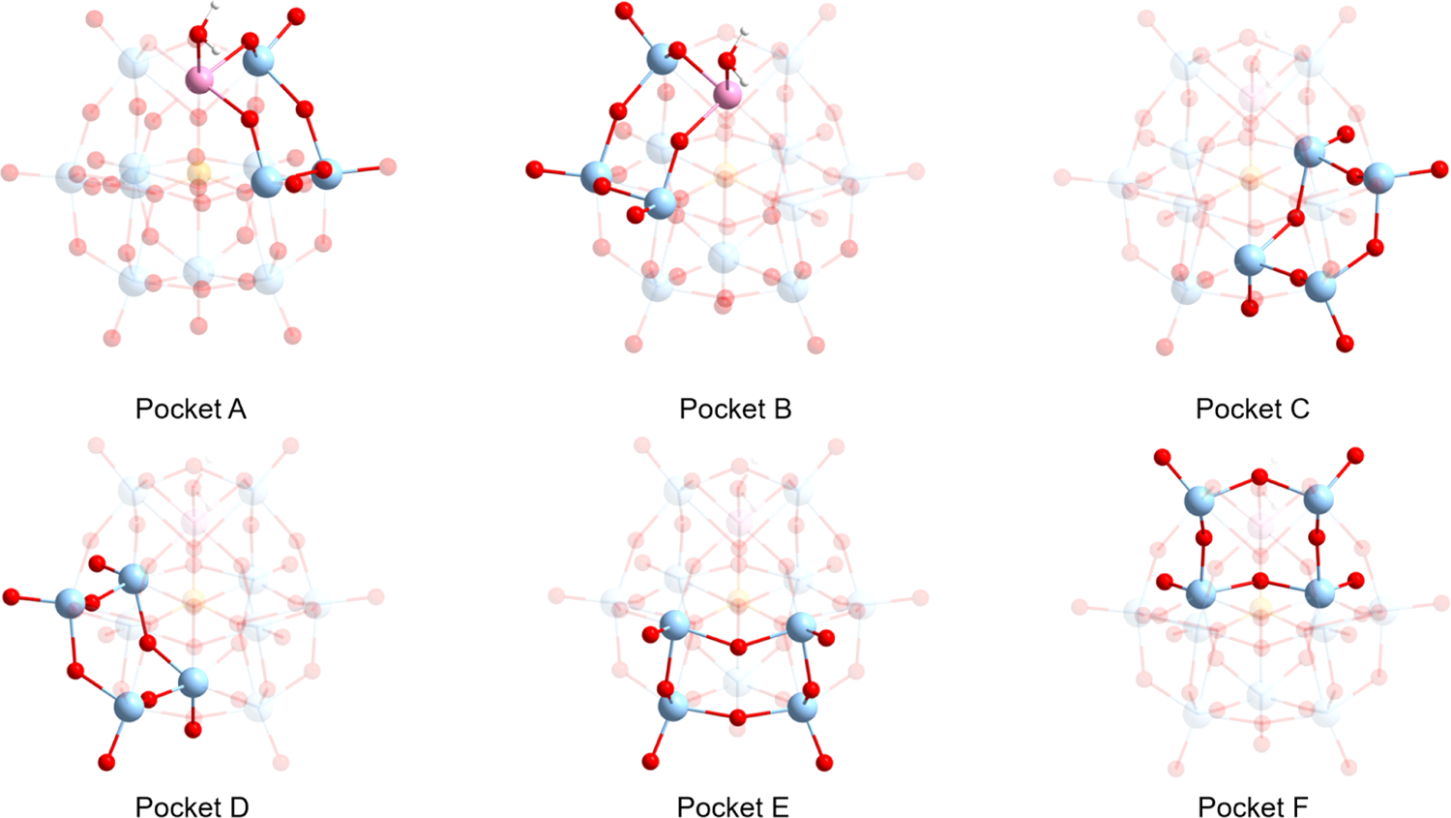
Schematic representations of 4-fold pockets (A–F) on the surface of [PW_11_Co(H_2_O)O_39_]^5–^ anions.

On the surface of the molecule, the counterion can interact with 4-fold (pocket A–F) or 3-fold pockets—see Figure 6. The 4-fold pockets feature four oxygen atoms capable of interacting with counterions, while 3-fold pockets are comprised three adjacent oxygen atoms which assume a triangular shape. In this work, we concentrated on counterion interactions with the 4-fold pockets, which bind significantly stronger than the 3-fold pockets due to improved Coulomb interactions.

To differentiate between cation arrangements, isomers were identified by their unoccupied pockets. For example, isomer-D corresponds to systems with an absent cation–oxygen interaction at pocket D—see Figure S3. The relative stability of all isomers was evaluated by the difference in electronic energy, *E*_Rel_.—see Table 2. Δ*E*_Rel_ across K_5_[PW_11_Co(H_2_O)O_39_] isomers were almost negligible at 1.11 kcal mol^–1^. The discrepancy between pocket A and B was attributed to reduced electrostatic attractive forces because of shielding by the aqua protons, correlating with previous work.^43^ Energy differences across K_4_[PW_11_Co(H_2_O)O_39_] isomers were moderate, reporting Δ*E*_Rel_ of 3.04 kcal mol^–1^. It is important to recognize the electronic energy difference between the three lowest energy isomers of K_4_[PW_11_Co(H_2_O)O_39_] ranged by >0.4 kcal mol^–1^. Hence, it is reasonable to assume all arrangements will co-exist in solution. However, for our redox calculations, we focused on the two lowest energy isomers: A and A,D.

**Table 2:** Relative Stability of Cation Arrangements on the Surface of the Keggin K_*x*_[PW_11_Co(H_2_O)O_39_]^*q*−*x*^ (*X* = 4 or 5) Salts^a^

pocket	*E*_Rel_
A	1.105
B	0.941
C	0.373
D	0.000
E	0.323
F	0.001
A,B	1.386
A,C	0.134
A,D	0.000
A,E	0.837
A,F	0.398
B,C	2.111
B,D	2.377
B,E	3.042
B,F	2.550
C,D	1.075
C,E	1.639
C,F	1.211
D,E	1.305
D,F	1.739
E,F	1.650

a All energies are reported in kcal mol^–1^.

**Figure 7:**
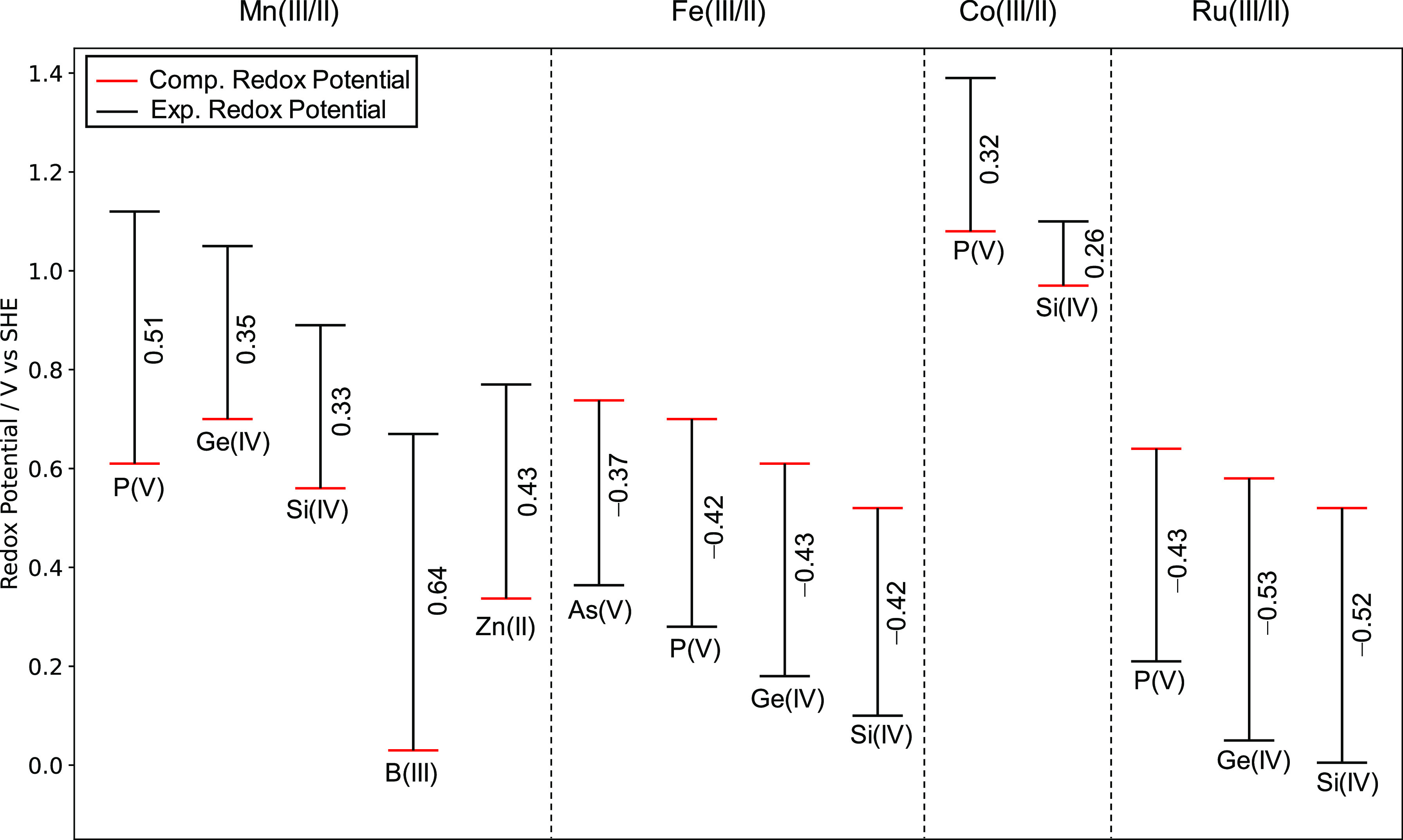
Redox potentials, *U*^0^_Red_ vs SHE, for Mn(III/II), Fe(III/II), Co(III/II), and Ru(III/II) couple present in K_*x*_[XW_11_M(H_2_O)O_39_]^*q*−*x*^; X = P(V), Si(IV), Ge(IV), B(III), and Zn(II). All calculations were performed with the A and A,D cation arrangements. All potentials were calculated using the PBE/TZP level of theory. Experimental potentials were obtained from Mn(III/II),^20^ Fe(III/II),^21^ Co(III/II),^23,24^ and Ru(III/II).^24,26,27^

Redox potentials, *U*^0^_Red_ versus SHE, for Mn(III/II), Fe(III/II), Co(III/II), and Ru(III/II) couples present in K_*x*_[XW_11_M(H_2_O)O_39_]^*q*−*x*^; X = As(V), Si(IV), Ge(IV), B(III), and Zn(II) are reported in Figure 7. All potentials were calculated using the PBE/TZP level of theory to allow for comparison with the anionic model, shown in Figure 4. The incorporation of counterions positively shifted redox potentials by >500 mV. Computation of *U*^0^_Red_ with K_*x*_[XW_11_M(H_2_O)O_39_]^*q*−*x*^ M = Mn(III/II)/Co(III/II) demonstrated significant improvement, by which *U*^0^_Error_ rarely exceeded 0.50 V. A comparison with Figure 4 shows *U*^0^_Error_ in K_*x*_[ZnW_11_Mn(H_2_O)O_39_]^*q*−*x*^ was reduced from 1.26 to 0.43 V (Δ*U*^0^_Erro__r_ = 0.83 V). However, accuracy in computation of *U*^0^_R__ed_ for K_*x*_[XW_11_M(H_2_O)O_39_]^*q*−*x*^ M = Fe(III/II)/Ru(III/II) was impaired in which all couples produced *U*^0^_Error_ exceeding 0.35 V.

**Figure 8:**
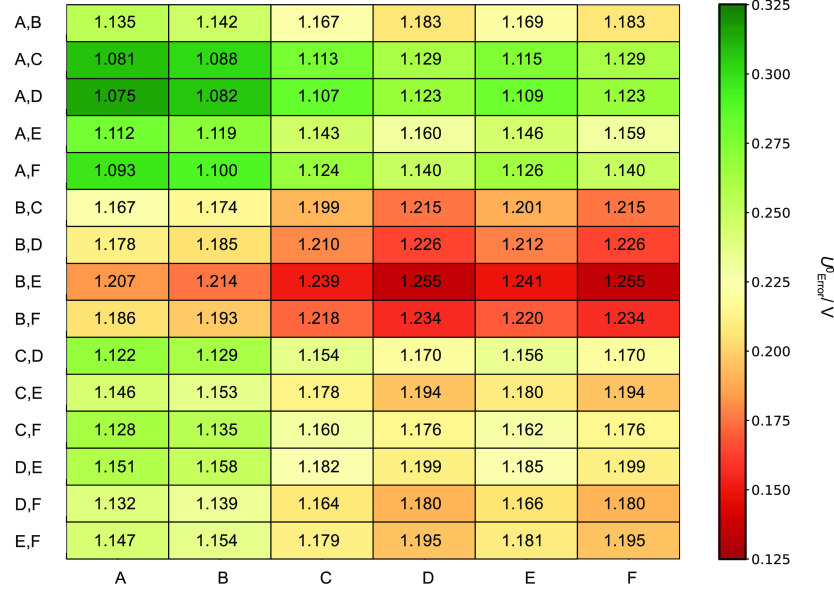
Redox potentials, *U*^0^_Red_ vs SHE, for all cation rearrangements for K_*x*_[PW_11_Co(H_2_O)O_39_]^*q*−*x*^; *x* = 4 or 5, obtained using the PBE/TZP methodology. Gibbs free energies for all cation arrangements were computed using the zero-point energies and entropic components obtained from GGA–vibrational frequencies for A and A,D isomers. Experimental potentials were obtained from Co(III/II).^23,24^

Thus far, models of K_*x*_[XW_11_M(H_2_O)O_39_]^*q*−*x*^ have employed the A and A,D isomers—see Table 2. Figure 8 reports *U*^0^_Error_ for K_*x*_[PW_11_Co(H_2_O)O_39_]^*q*−*x*^ to assess the sensitivity of our model to different cation arrangements. The current model demonstrated moderate sensitivity across all cation arrangements ranging up to 0.18 V. Comparatively poor agreement with the literature was achieved with the energetically favored A + A,D couples, whose redox couple underestimated potentials by 0.315 V, with respect to the literature. Accurate computation of *U*^0^_Red_ was achieved with isomers: D + B,E and F + B,E couples, who redox couples underestimated potentials by 0.135 V.

**Figure 9:**
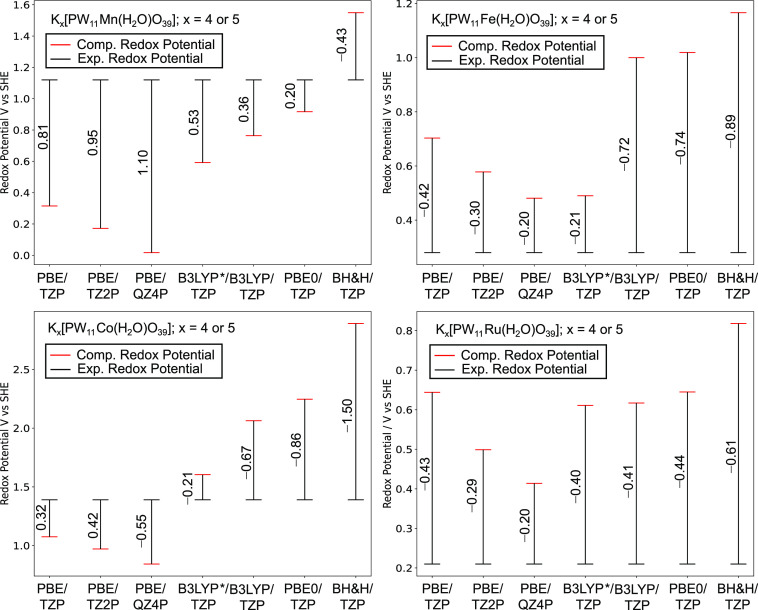
Redox potentials, *U*^0^_Red_ vs SHE, for Mn(III/II), Fe(III/II), Co(III/II), and Ru(III/II) redox couples in K_*x*_[PW_11_M(H_2_O)O_39_]^*q*−*x*^. All calculations were performed with the A and A,D cation arrangements. Experimental potentials were obtained from Mn(III/II),^20^ Fe(III/II),^21^ Co(III/II),^23,24^ and Ru(III/II).^24,26,27^

For comparison with Figure 5, we have explored the effect of applied *x*–*c* functional and basis set on *U*^0^_R__ed_ for K_*x*_[PW_11_M(H_2_O)O_39_]^*q*−*x*^ M = Mn(III/II), Fe(III/II), Co(III/II), and Ru(III/II), as shown in Figure 9. The employment of larger basis sets (TZ2P, QZ4P) positively shifted potentials for Mn(III/II) and Co(III/II) couples providing poorer reproduction of the literature, compared to triple-ζ + polarization (TZP) basis sets. This effect was particularly pronounced for K_*x*_[PW_11_M(H_2_O)O_39_]^*q*−*x*^ in which *U*^0^_E__rror_ increased from 0.35 to 1.07 V (Δ*U*^0^_E__rror_ = 0.72 V). A comparison with Figure 5 shows incorporation of counterions to [PW_11_Mn(H_2_O)O_39_]^*q*−^ reduced *U*^0^_Error_ by 0.68, 0.24, and 0.33 V for TZP, TZ2P, and QZ4P basis sets, respectively.

We have explored the effect of HF exchange on *U*^0^_Red_ for K_*x*_[PW_11_M(H_2_O)O_39_]^*q*−*x*^ M = Mn(III/II), Fe(III/II), Co(III/II), and Ru(III/II). Δ*U*^0^_E__rror_ using GGA–PBE functional (0% exchange) reflected in the range of 0.32–0.43 V across all redox couples. Those with larger contributions of HF exchange varied significantly, for example, Δ*U*^0^_Er__ror_ computed using BH&H ranged from 0.43 to 1.50 V. Hybrid functionals exceeding 25% HF exchange did not provide any improvement for reducing Δ*U*^0^_Error_. As is evident, increasing HF exchange positively shifted *U*^0^_Red_ which was attributed to the over-stabilization of the ion-pairs. Hybrid functionals exceeding 25% HF are not recommended because of large CPU times coupled with significant overestimations to Δ*U*^0^_Red_.

**Figure 10:**
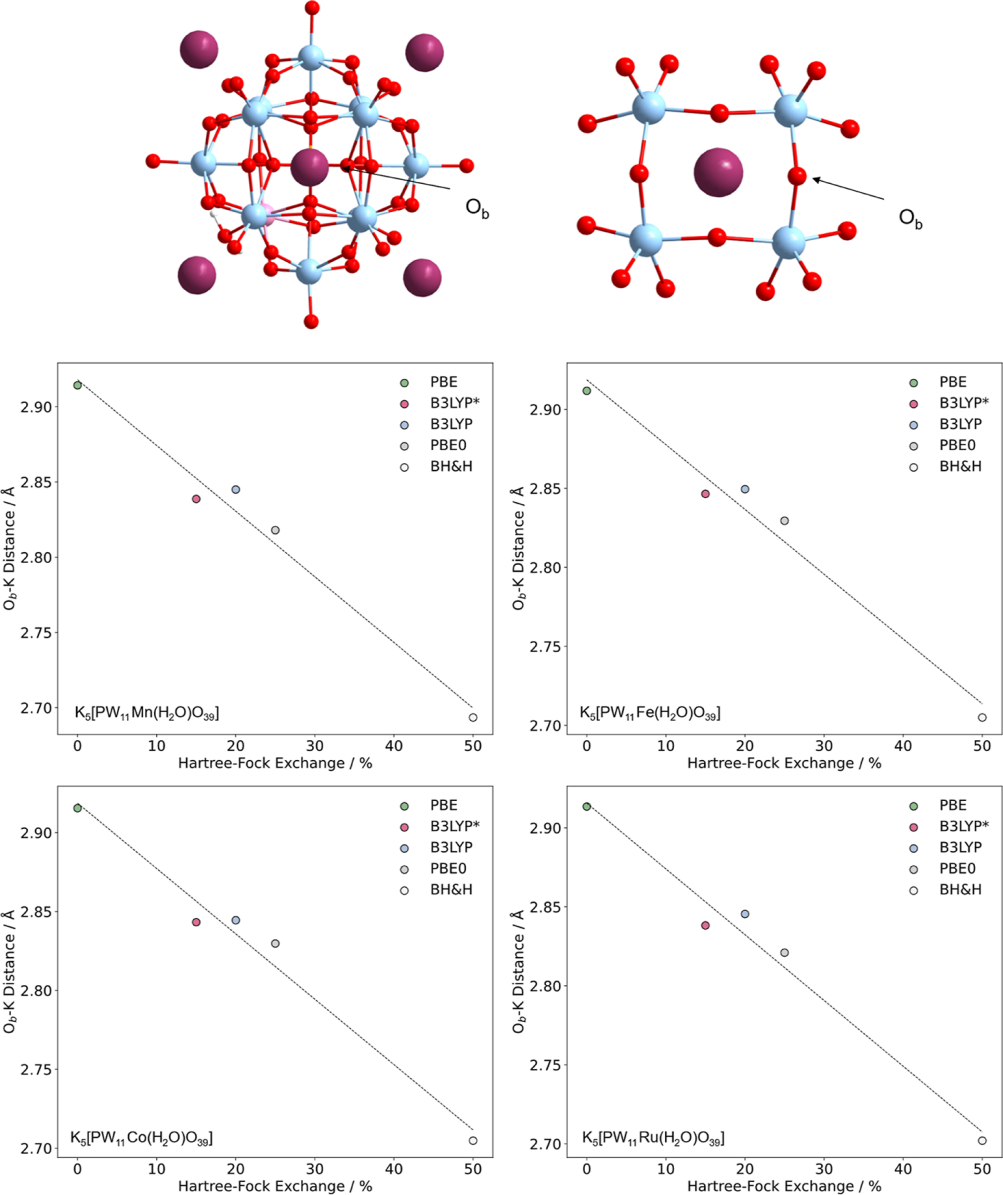
Average counterion–bridging oxygen distance (O_b_–K) in K_5_[PW_11_M(H_2_O)O_39_], where M = Mn(II), Fe(II), Co(II), and Ru(II) plotted as a function of HF exchange. All calculations were performed with the A and A,D cation arrangements.

*U*^0^_Red_ became positively shifted with the increase in contributions of HF exchange. To rationalize this observation, we plotted counterion–bridging oxygen (between pocket D) as a function of HF exchange, see Figure 10. Increasing contributions of HF exchange produced shorter O_b_–K distances leading to the over-stabilization of the close contact ion-pairs. The significance of these O_b_–K distances is emphasized by the computed range (PBE to BH&H) in O_b_–K distances in K_5_[PW_11_Co(H_2_O)O_39_] calculated at ca. 0.2 Å which reflected in Δ*U*^0^_Red_ = 1.29 V. Previous work by Kaledin and co-workers reported the average O_t_–K and O_b_–K distances for hydrated [PW_12_O_40_][K(H_2_O)_16_]_3_ complexes were 5.1 and 5.7 Å, respectively.^63^ To contrast, O_t_–K and O_b_–K distances in K_5_[PW_11_Co(H_2_O)O_39_] were ca. 4.1 and 2.8 Å, respectively. Evidently, further improvements should focus on accurately modeling counterion–bridging oxygen distances to enable more accurate computation of *U*^0^_Red_.

## Conclusions

In this work, we have employed DFT calculations to systematically study the accuracy of various exchange–correlation functionals for reproducing experimental redox properties in mono-transition-metal-substituted Keggin [XW_11_M(H_2_O)O_39_]^*q*−^ anions and their corresponding potassium salts. We have focused our attention to Mn(III/II), Fe(III/II), Co(III/II), and Ru(III/II) redox couples associated with [XW_11_M(H_2_O)O_39_]^*q*−^ X = P(V), Si(IV), Ge(IV), B(III), and Zn(II). Our aim has been to explore the challenges in computing redox potentials and provide an insight into the geometric and electronic factors controlling it. We employed several *x*–*c* functionals including the hybrid class selected by their contributions to HF exchange (15% B3LYP*, 20% B3LYP, 25% PBE0, and 50% BH&H).

For direct comparison, we explicitly located K^+^ counterions to render our system charge neutral to reduce systemic discrepancies associated with the self-interaction error. The incorporation of counterions positively shifted redox potentials by >500 mV. By incorporating counterions, significant improvement to *U*^0^_Red_ for Mn(III/II) and Co(III/II) redox couples, in which *U*^0^_Error_ rarely exceeded 0.50 V. This effect was particularly pronounced for *U*^0^_Error_ in K_*x*_[ZnW_1__1_Mn(H_2_O)O_39_]^*q*−*x*^, whereby *U*^0^_Error_ was reduced from 1.26 to 0.43 V (Δ*U*^0^_Error_ = 0.83 V). However, problems remain as *U*^0^_Red_ for Fe(III/II) and Ru(III/II) were excessively (positively) shifted, attributed to the over-stabilization of the ion-pairs. Previous work has shown the average O_t_–K and O_b_–K distances for hydrated [PW_12_O_40_][K(H_2_O)_16_]_3_ complexes were 5.1 and 5.7 Å, while our charge neutral model of K_5_[PW_11_Co(H_2_O)O_39_] produced O_t_–K and O_b_–K distances reporting at ca. 4.1 and 2.8 Å, respectively. We have rationalized this overestimation by plotting O_b_–K distances as a function of HF, whereby increasing contributions of exchange (0% PBE to 50% BH&H) produced progressively shorter O_b_–K distances. The reported range in O_b_–K was only ca. 0.2 Å; however, its significance is highlighted in K_5_[PW_11_Co(H_2_O)O_39_] which reflected in the range of 1.29 V for *U*^0^_Red_.

Obtaining accurate redox potentials using implicit solvation models remains a challenge. Our results emphasize that understanding the nature of the electrode and electrolyte environment may be essential to obtaining reasonable agreement between theoretical and experimental results. Further improvements to this work by accurately modeling counterion–bridging oxygen distance will enable more accurate computation of *U*^0^_Red_. The present results emphasis the current approach requires significant improvement to achieve more reliable modeling with respect to experimental work.

## Supporting Information Available

Optimized geometries for [PW_11_M(H_2_O)O_39_]^*q*−^ and A and A,D isomers of K_*x*_[PW_11_M(H_2_O)O_39_]^*q*−*x*^ (*x* = 4 or 5); schematic molecular orbital (MO) diagram for Mn(III/II), Fe(III/II), Co(III/II), and Ru(III/II) couples in [XW_11_M(H_2_O)O_39_]^*q*−^, where X = As(V), Si(IV), Ge(IV), B(III), and Zn(II); schematic MO for [PW_11_M(H_2_O)O_39_]^*q*−^ computed with several *x*–*c* functionals; and MO diagrams for K_*x*_[PW_11_M(H_2_O)O_39_]^*q*−*x*^ (*x* = 4 or 5) obtained from the PBE/TZP level of theory (PDF)

## Notes

The data sets generated using DFT calculations are available in the ioChem-BD database (https://doi.org/10.19061/iochem-bd-6-254).
