# Supplementary material for: Reducing Systematic Uncertainty in Computed Redox Potentials for Aqueous Transition-Metal-Substituted Polyoxotungstates
Source: Inorg Chem. 2023 Jul 25;62(31):12260–71. doi: 10.1021/acs.inorgchem.3c01115 (PMC10410613; doi:10.1021/acs.inorgchem.3c01115)
Supplement: Supplementary file 1 — ic3c01115_si_001.pdf [file ic3c01115_si_001.pdf]

Supporting Information

Reducing Systematic Uncertainty in Computed Redox Potentials for Aqueous Transition−Metal−Substituted Polyoxotungstates

Jake A. Thompson[1], Rebeca González−Cabaleiro[2], Laia Vilà−Nadal[1]**

[1]*School of Chemistry, University of Glasgow, Glasgow, United Kingdom*

[2]*Department of Biotechnology, Delft University of Technology, Delft, Netherlands*

*Email: R.GonzalezCabaleiro@tudelft.nl & laia.vila−nadal@chem.gla.ac.uk

Table SI-1: Optimized geometries of $[PW_{11}M(H_2O)O_{39}]^{q-}$ performed using PBE / TZP. Bond lengths and angles reported in Å and °, respectively.

$[PW_{11}M(H_2O)O_{39}]^{q-}$	O_c–P	O_c–M	O_t–M	O_{a1}–M	O_{b2}–M	O_{a1}–W	O_{b2}–W	O_{a1}–M–O_{b2}
$[PW_{11}Mn(H_2O)O_{39}]^{4-}$	1.555	2.302	2.338	1.940	1.923	1.880	1.868	173.2
$[PW_{11}Mn(H_2O)O_{39}]^{5-}$	1.558	2.377	2.303	2.137	2.048	1.829	1.833	168.0
$[PW_{11}Fe(H_2O)O_{39}]^{4-}$	1.565	2.236	2.179	2.021	1.963	1.859	1.864	171.3
$[PW_{11}Fe(H_2O)O_{39}]^{5-}$	1.562	2.275	2.226	2.047	2.013	1.840	1.833	170.8
$[PW_{11}Co(H_2O)O_{39}]^{4-}$	1.586	1.964	1.976	1.910	1.917	1.877	1.844	177.1
$[PW_{11}Co(H_2O)O_{39}]^{5-}$	1.561	2.248	2.202	2.072	2.013	1.831	1.824	173.5
$[PW_{11}Ru(H_2O)O_{39}]^{4-}$	1.579	2.129	2.151	2.029	2.028	1.881	1.859	178.3
$[PW_{11}Ru(H_2O)O_{39}]^{5-}$	1.576	2.149	2.166	2.066	2.031	1.849	1.831	177.4

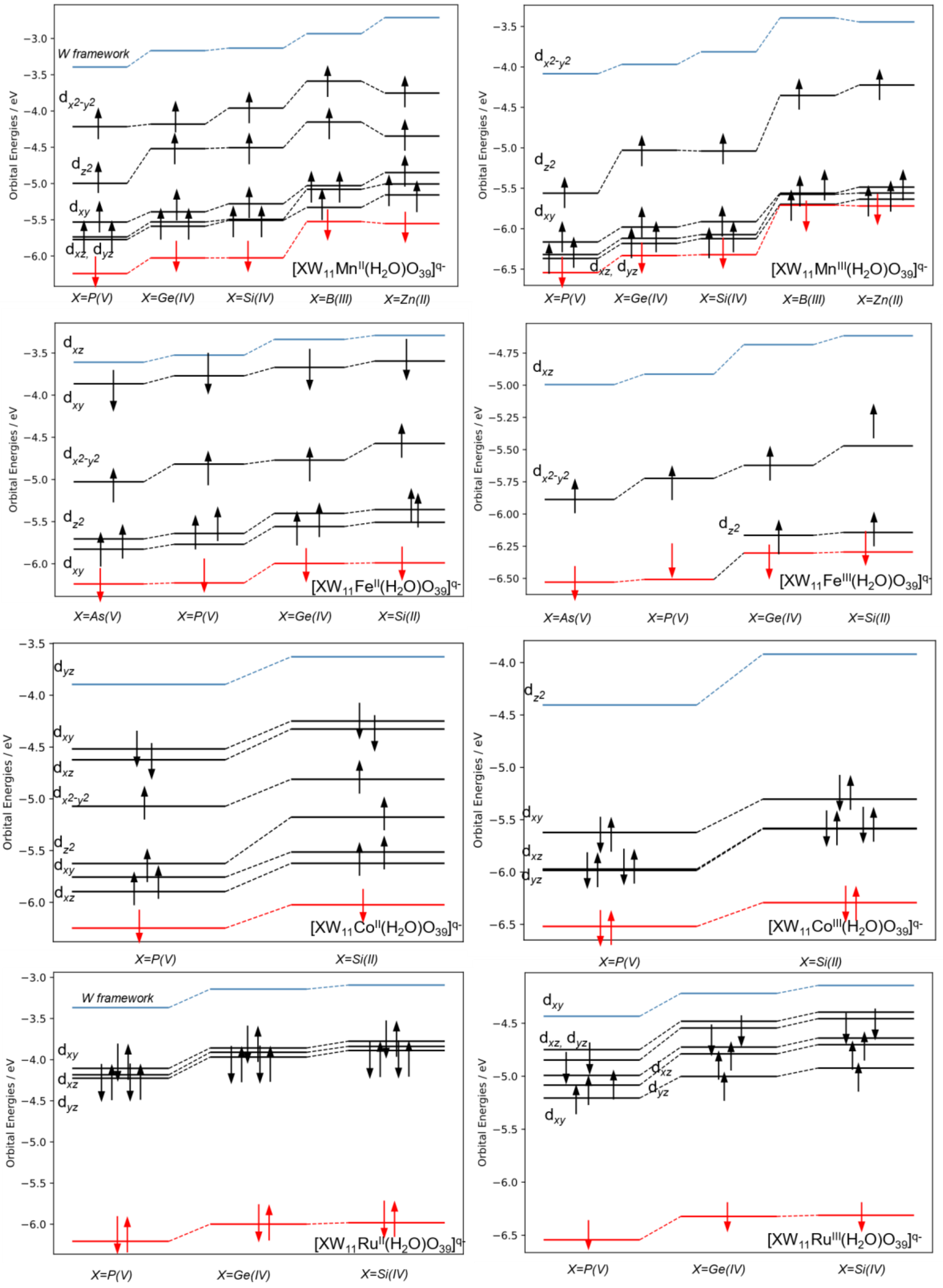

Figure SI-1: Schematic molecular orbital diagram for Mn(III/II), Fe(III/II), Co(III/II), and Ru(III/II) couples present in $[XW_{11}M(H_2O)O_{39}]^{q-}$; X= As(V), Si(IV), Ge(IV), B(III), Zn(II) calculated at the PBE/TZP level of theory. All energies were reported in eV.

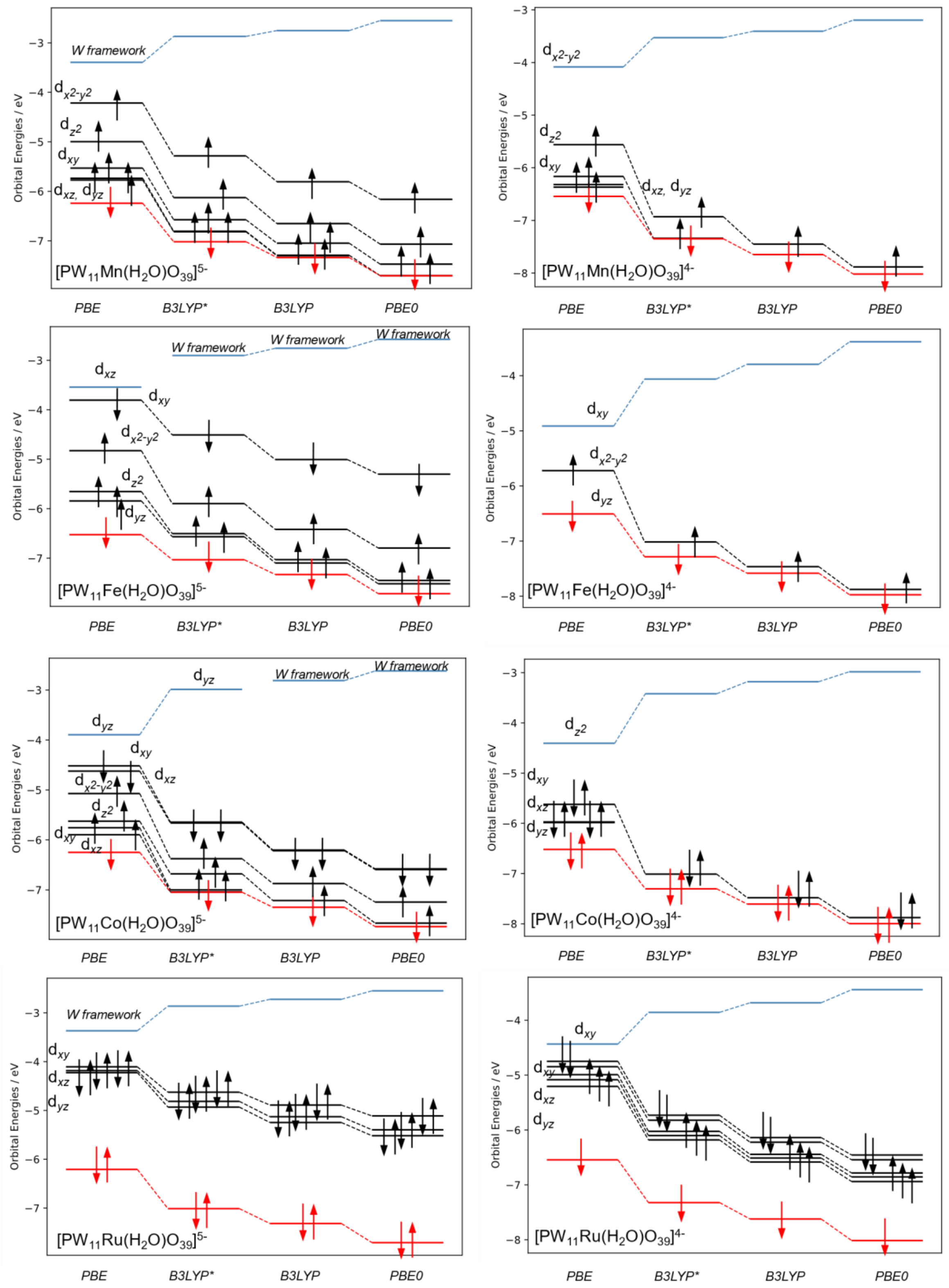

Figure SI-2: Schematic molecular orbital diagram for Mn(III/II), Fe(III/II), Co(III/II), and Ru(III/II) couples present in $[PW_{11}M(H_2O)O_{39}]^{q-}$ performed using GGA-PBE and hybrid functionals selected on their respective contributions of Hartree-Fock (HF) exchange (15 % B3LYP*, 20 % B3LYP, 25 % PBE0, and 50 % BH&H).

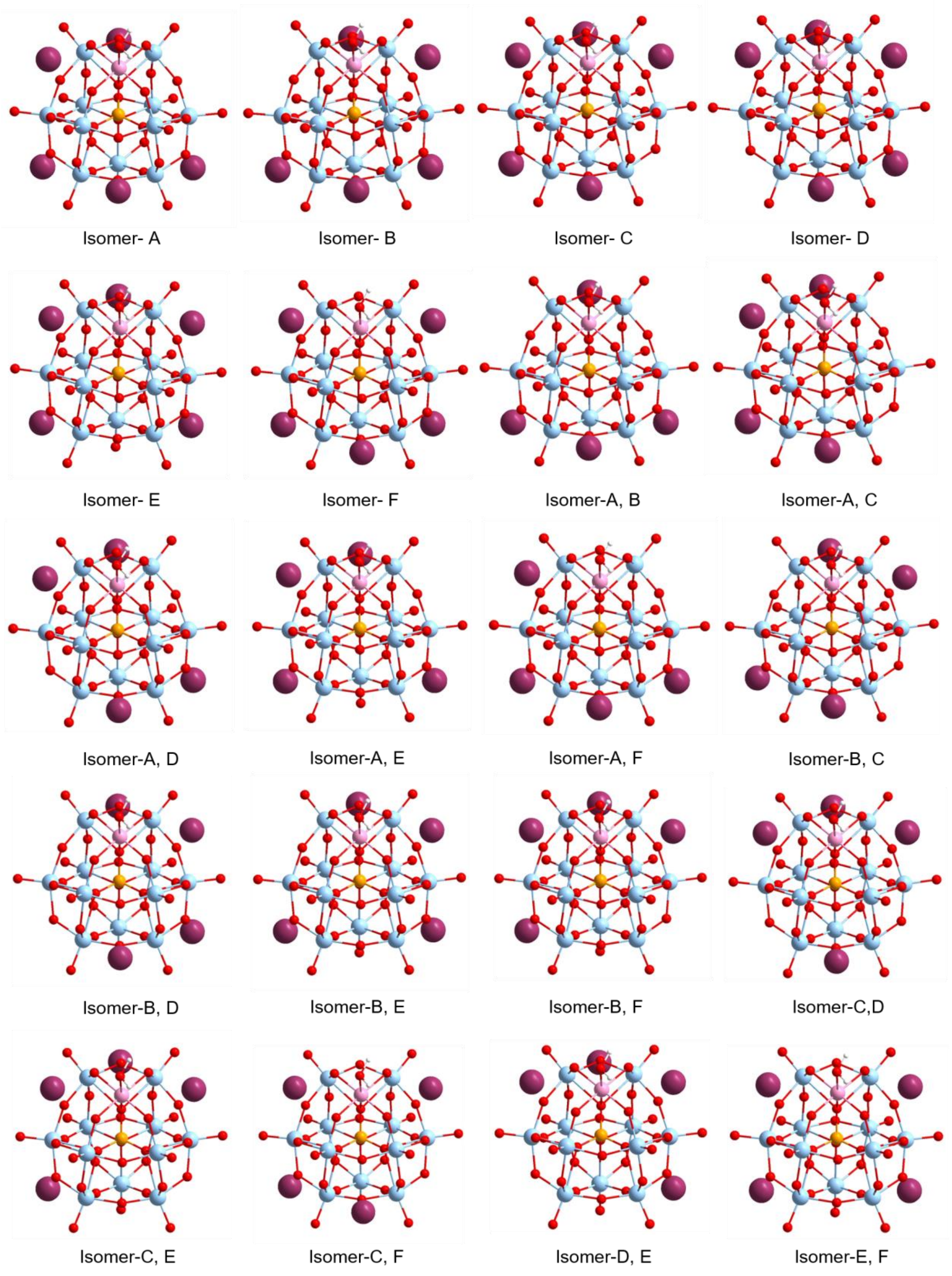

Figure SI-3: All cation arrangements for $K_x[PW_{11}M(H_2O)O_{39}]$; x = 4 or 5. The isomers were labelled based on vacancies. For example, isomer−D corresponds to the isomer without a cation−oxygen interaction at pocket D.

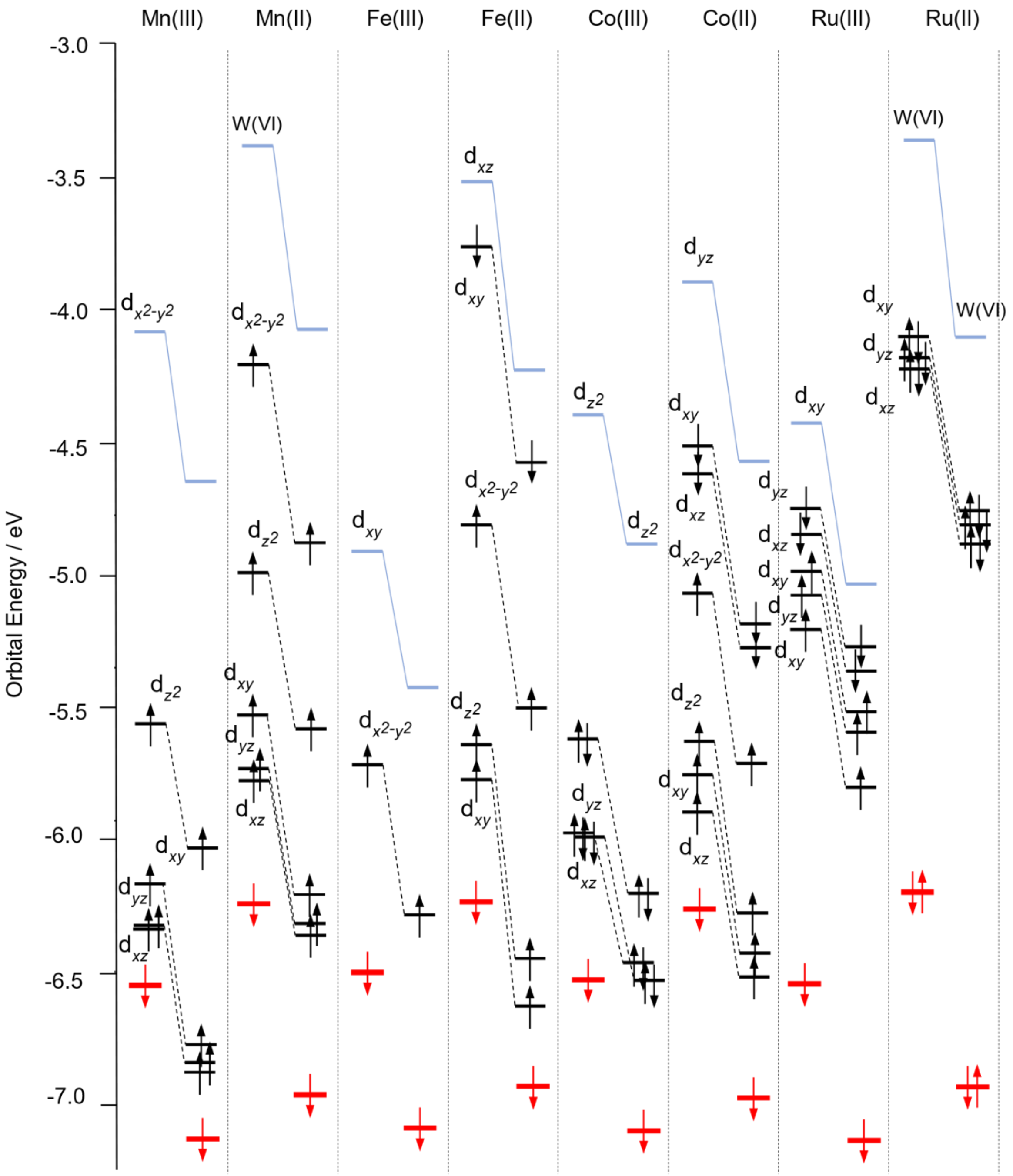

Figure SI-4: Schematic molecular orbital diagram for $[PW_{11}M(H_2O)O_{39}]^{q-}$ (left) and corresponding $K_x[PW_{11}M(H_2O)O_{39}]^{q-x}$; x = 4 or 5 (right) optimized using PBE/TZP level of theory. Colours correspond to red = O2(p), blue = W, and black = transition−metal. All orbital energies reported in eV.

Table SI-2: Optimized geometric parameters of the A and A,D isomers in $K_x[PW_{11}M(H_2O)O_{39}]^{q-x}$; x = 4 or 5 performed using PBE / TVP. Bond lengths and angles reported in Å and °, respectively.

$K_x[PW_{11}M(H_2O)O_{39}]^{q-x}$	O_c–P	O_c–M	O_t–M	O_{a1}–M	O_{b2}–M	O_{a1}–W	O_{b2}–W	O_{a1}–M–O_{b2}
$K_4[PW_{11}Mn(H_2O)O_{39}]$	1.557	2.414	2.258	2.167	2.039	1.831	1.825	166.8
$K_5[PW_{11}Mn(H_2O)O_{39}]$	1.554	2.316	2.292	1.949	1.920	1.891	1.860	172.7
$K_4[PW_{11}Fe(H_2O)O_{39}]$	1.564	2.256	2.150	2.027	1.955	1.868	1.858	170.6
$K_5[PW_{11}Fe(H_2O)O_{39}]$	1.562	2.302	2.162	2.099	1.996	1.837	1.824	169.4
$K_4[PW_{11}Co(H_2O)O_{39}]$	1.585	1.967	1.966	1.919	1.913	1.880	1.843	177.1
$K_5[PW_{11}Co(H_2O)O_{39}]$	1.560	2.251	2.151	2.067	2.066	1.839	1.829	173.4
$K_4[PW_{11}Ru(H_2O)O_{39}]$	1.578	2.131	2.140	2.036	2.018	1.891	1.851	177.0
$K_5[PW_{11}Ru(H_2O)O_{39}]$	1.574	2.153	2.150	2.077	2.019	1.856	1.825	177.0

Analysis for the selected structural parameters for the A and A,D isomers in $K_x[PW_{11}M(H_2O)O_{39}]^{q-x}$ are tabulated in Table SI−2. The frontier molecular orbitals of potassium, $K_x[PW_{11}M(H_2O)O_{39}]^{q-x}$ salts presented in Figure SI−4 will be employed to rationalize any discrepancies in geometry following redox processes. As is evident, significant constriction of O_c−M bond of 0.284 Å is induced following one−electron oxidation of $K_5[PW_{11}Co(H_2O)O_{39}]$. To contrast, one−electron oxidations of $[PW_{11}Co(H_2O)O_{39}]^{5-}$ constricted O_c−M by 0.284 Å to the anionic model. Evidently, the structural integrity of the POM is maintained following the incorporation of counterions.

Table SI-3: Optimized bond parameters for all cation arrangements for $K_x[PW_{11}M(H_2O)O_{39}]^{q-x}$; x = 4 or 5.

Hole	O_c–P	O_c–M	O_t–M	O_{a1}–M	O_{b2}–M	O_{a1}–W	O_{b2}–W	O_{a1}–M–O_{b2}
A	1.560	2.256	2.150	2.076	2.044	1.837	1.811	173.4
B	1.560	2.280	2.146	2.045	2.076	1.822	1.826	172.2
C	1.559	2.243	2.153	2.066	2.071	1.839	1.827	174.4
D	1.561	2.251	2.151	2.067	2.066	1.839	1.829	173.8
E	1.561	2.248	2.154	2.070	2.060	1.838	1.831	174.1
F	1.561	2.248	2.155	2.069	2.060	1.838	1.829	174.0
**Hole**	**O_c–P**	**O_c–M**	**O_t–M**	**O_{a1}–M**	**O_{b2}–M**	**O_{a1}–W**	**O_{b2}–W**	**O_{a1}–M–O_{b2}**
A,B	1.584	1.971	1.964	1.910	1.915	1.860	1.842	177.2
A,C	1.586	1.967	1.966	1.921	1.911	1.878	1.844	177.0
A,D	1.585	1.967	1.966	1.919	1.913	1.880	1.843	177.1
A,E	1.585	1.969	1.965	1.921	1.912	1.877	1.846	176.9
A,F	1.583	1.966	1.966	1.921	1.913	1.881	1.842	176.9
B,C	1.586	1.966	1.968	1.907	1.928	1.861	1.863	176.9
B,D	1.586	1.966	1.968	1.906	1.931	1.863	1.863	176.9
B,E	1.587	1.967	1.967	1.907	1.932	1.861	1.866	176.7
B,F	1.584	1.965	1.969	1.907	1.931	1.863	1.861	176.7
C,D	1.587	1.963	1.969	1.916	1.927	1.880	1.864	176.9
C,E	1.588	1.963	1.970	1.918	1.927	1.878	1.867	176.6
C,F	1.585	1.961	1.971	1.918	1.926	1.881	1.863	176.6
D,E	1.588	1.964	1.969	1.917	1.930	1.880	1.867	176.7
D,F	1.585	1.962	1.970	1.917	1.929	1.883	1.863	176.7
E,F	1.585	1.962	1.970	1.919	1.929	1.880	1.866	176.5

We have explored the structural sensitivity of $K_x[PW_{11}Co(H_2O)O_{39}]^{q-x}$ (x = 4 or 5) across all configurations shown in Figure SI−3. As previously discussed, the minute energy difference between configurations suggests spontaneously inter−conversion in solution. The axial bonding in $K_5[PW_{11}Co(H_2O)O_{39}]$ represented by the O_c–M and O_t–M averaged at 2.254 and 2.152 Å, respectively. The O_c–M parameter was sensitive to alteration in cation arrangement, ranging by up to 0.037 Å. By contrast, terminal oxygen bonding (O_t) remained stable across ranging by 0.009 Å. Average equatorial distances in $K_5[PW_{11}Co(H_2O)O_{39}]$ corresponding to O_{a1}–M and O_{b2}–M were calculated at 2.066 and 2.063 Å, respectively. Across all arrangements, equatorial bonding ranged by 0.031 and 0.032 Å for O_{a1}–M and O_{b2}–M, respectively, highlighting the sensitivity of the inner coordination sphere localized around the incorporated heteroatom. The range across all arrangements for O_{a1}–W and O_{b2}–W was

0.017 and 0.020 Å, reflecting lower than the corresponding heteroatom−oxygen bridges. The structural sensitivity across all arrangements of $K_4[PW_{11}Co(H_2O)O_{39}]$ was significantly smaller than the previous. Generally, the inner coordinating sphere at the heteroatom was maintained; discrepancies did not exceed 0.021 Å that were observed in O_{b2}−M. The largest discrepancies were observed for the O_{a1}−W and O_{b2}−W parameters at 0.023 and 0.025 Å, respectively.
